# Influence of MXene Particles with a Stacked-Lamellar Structure on Osteogenic Differentiation of Human Mesenchymal Stem Cells

**DOI:** 10.3390/ma14164453

**Published:** 2021-08-09

**Authors:** Jun-Hwee Jang, Eun-Jung Lee

**Affiliations:** Department of Nano-Biomedical Science, BK21 PLUS NBM Global Research Center for Regenerative Medicine, Dankook University, Cheonan 31116, Korea; junhweej@dankook.ac.kr

**Keywords:** MXene, intercalation, two-dimensional, stacked-lamellar, osteogenic differentiation

## Abstract

MXenes with a two-dimensional (2D) structure have attracted attention as potential biomedical materials. In this study, Ti_3_C_2_ MXene particles with 2D-lamellar structures were intercalated and their potential as a biomaterial was evaluated using human mesenchymal stem cells. Intercalated MXene was characterized in terms of microstructure, phase composition, and size. Cell proliferation experiments with MXene particles confirmed that concentrations >50 μg/mL were cytotoxic, while concentrations <20 μg/mL promoted osteogenic differentiation. Moreover, MXene effectively facilitated the early and late osteogenic gene expression.

## 1. Introduction

Many pathways have led to the discovery of new materials with completely new properties. Bioceramics are very useful in hard-tissue engineering and have structural stability and superior biocompatibility [1]. Traditional industrial oxides, such as zirconia, alumina, and titanium oxide, have been applied biologically when new biological properties have been discovered [2,3,4].

MXenes were discovered in 2011. They are hydrophilic two-dimensional (2D) materials that have been studied in various applications, such as sensors, catalysis, and water purification [5,6,7,8]. Their use as biomedical materials has attracted attention, especially their antibacterial effects, photothermal-conversion efficiency, and fluorescence properties [9]. They are expected to show good biostability compared to 2D graphene-based materials due to their biodegradability [10,11]. MXenes naturally form stacked structures, which increase the surface available for drug attachment and reduce cell damage induced by direct contact with 2D MXene, compared to MXene nanosheets [12,13,14]. This stacked-lamellar structure can enhance the application of MXene particles as drug carriers [10].

Despite the interest as a biomaterial, little is known about the interactions of MXenes with stem cells or their biological tissue affinity [10]. MXenes have been applied as bone regeneration composites due to their excellent mechanical properties for tissue engineering [15,16]. However, there are no reports on the osteo-inductive or -conductive properties of MXene particles or flakes. As a potential biomaterial, it is essential to investigate their biological characteristics for wide application in hard-tissue engineering [17,18].

In this study, MXene particles with a stacked 2D-flake structure were prepared by intercalation, resulting in a more favorable interlocking structure for producing composites or incorporating drugs [19]. The effects of the MXenes on the bone differentiation behavior of human mesenchymal stem cells were also evaluated [20,21].

## 2. Materials and Methods

Ti_3_C_2_ MXene (Invisible, Korea) was proceeded through intercalation at room temperature. MXene particles were mixed with tetramethylammonium hydroxide for 3 days, and the morphology of the intercalated MXene was observed using field emission scanning electron microscopy (FE-SEM; Sigma300, Zeiss, Oberkochen, Germany). The particle size was investigated using Zetasizer (Nano-ZS Zetasizer, Malvern Panalytical, Malvern, UK) and the phase composition by X-ray diffraction (XRD; Ulima IV, Rigaku, Tokyo, Japan). Intercalated MXene was used throughout all experiments in this study.

The cytotoxicity of MXene was evaluated in human mesenchymal stem cells (hMSCs, A15652, Thermo Fischer, Waltham, MA, USA) treated with MXene at concentrations of 0–100 μg/mL and cultured for up to 7 days. Cell proliferation was assessed using the MTS kit (G3580, Promega, Madison, WI, USA) [22], and cell morphology was observed after culture for 1 day using confocal laser scanning microscopy (CLSM; Zeiss).

The alkaline phosphatase (ALP, Anaspec, Fremont, CA, USA) activity was measured after 7 and 14 days using *p*-nitrophenylphosphate, according to the manufacturer’s protocol [23].

Quantitative real-time polymerase chain reaction (qRT-PCR) was performed to investigate the osteogenic effects of MXene via gene expression. The synthesis of cDNA from total RNA was examined for 2 weeks in accordance with the protocol of the cDNA Synthesis Kit (NanoHelix, Daejeon, Korea) [24]. First, 1 μg of RNA extracted from cells was used to synthesize cDNA by reverse transcription. The expression of osteogenic markers was examined using SYBR green master mix (NanoHelix) [25], normalized to the expression of glyceraldehyde-3-phosphate dehydrogenase and calculated as 2^ΔΔCt^. The Alizarin Red S (ARS, Sigma-Aldrich, St. Louis, MO, USA) assay was used to detect calcium deposits generated by hMSC at 14 days, using normal (alpha-MEM, LM 008-02, Welgene, Gyeongsan, Korea) and osteogenic (O/M) media [26,27]. The mineralized cells stained red, and the amount of staining was quantified at 405 nm.

The quantitative results were performed at the least three replicates from each test group. All the results were presented as mean ± deviation. The statistical analyses were performed using a *t*-test and comparisons between groups were analyzed by one-way analysis of variance test. The differences with *p* < 0.05 were considered statistically significant (* *p* < 0.05, ** *p* < 0.01, *** *p* < 0.001).

## 3. Results and Discussion

**Figure 1:**
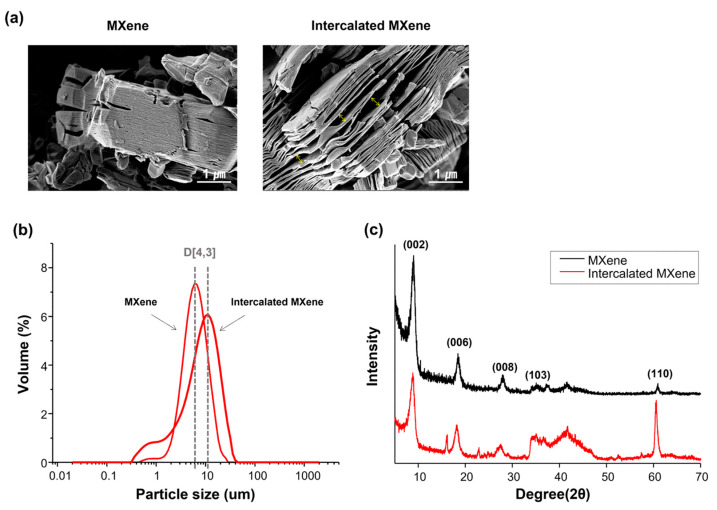
SEM images (**a**) and particle size distribution (**b**) of MXene before and after intercalation, and the XRD pattern of the MXenes (**c**).

Figure 1 shows the properties of intercalated MXene. The SEM images show the stacked-lamellar structure of the MXene. After intercalation, the gaps between 2D sheets of MXene were wider, and the average volume increased [28]. Figure 1b illustrates the size distribution graphs of MXene particles. D [3,4] indicates the mean size of particles based on volume. The average sizes of MXene and intercalated MXene particles were 6.3 and 11.2 μm, respectively. After the intercalation of MXene, XRD was used to determine whether a phase or crystallographic change had occurred. The XRD pattern in Figure 1c shows the typical crystallographic peaks of MXene before and after intercalation.

Intercalation is an intermediate step in the process of delaminating MXene with a stacked structure to a 2D lamella structure, which increases the effective surface area of MXene particles and, if necessary, adjusts the thickness of the MXene particles. The intercalation in this study is also performed because we aimed to assess MXene in a more favorable form for application to therapeutic agent loading or composite preparation. Intercalation is treated using intercalating compounds, such as DMSO [29,30,31], CTAB [32], alkylamines, isopropyl alcohol, urea, tetrapropylammonium hydroxide [33], and metal cations [34,35]. We conducted the intercalation using tetramethylammonium hydroxide, which allowed us to obtain intercalated MXene. After the intercalation treatment, the typical crystallographic peaks of MXene were detected through XRD, but the overall peaks were relatively broad, and the peak intensity of the (002) plane decreased compared to that of pristine MXene. The decrease in the (002) peak is caused by the decrease in the crystallinity of MXene [36]. Moreover, the intensity of the (110) plane increased relative to other characteristic peaks, which is attributed to structural expansion by the intercalation process [36]. This XRD pattern analysis demonstrates the successful preparation of intercalated MXene.

**Figure 2:**
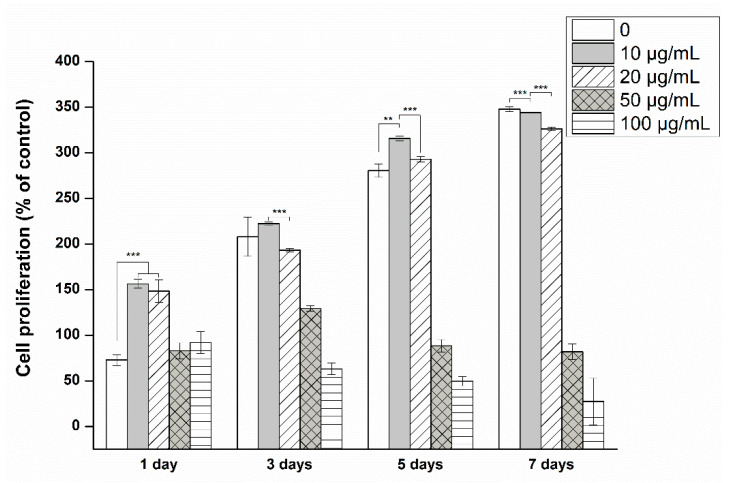
Cell proliferation at different MXene concentrations (** *p* < 0.01 and *** *p* < 0.001).

The effects of MXene at concentrations of 0–100 μg/mL on cytotoxicity and proliferation were assessed Figure 2. Cell proliferation on the first day was higher at low MXene concentrations (<20 μg/mL) compared to a tissue culture plate (TCP) without MXene. However, beginning day three, the proliferation on TCP and MXene were similar. Results after 5 days of incubation showed a significant decrease in cell proliferation at 50 μg/mL. Although 50 μ/mL is slightly cytotoxic, it does not strongly attack cells from scratch, such as a concentration of 100 μg/mL; thus, it seems that the cells, even weakly, survived and multiplied slightly until the third day. However, it is thought that the growth rate, after three days, of the remaining cells was very low because unhealthy cells were eliminated significantly during the culture media exchange after 3 days of culture. The hMSC population was reduced when exposed to >50 μg/mL MXene for 7 days, indicating cell toxicity. For all subsequent cell experiments, concentrations <50 μg/mL were used.

Many studies have been reported that cells maintain viability at concentrations ranging from tens to hundreds of μg/mL of MXenes [37,38,39]. Among them, some publications show the cytotoxicity result at a similar concentration level of MXene to this study [39]. Previous studies were conducted using various cell lines such as cancer cells and pre-osteoblast [40]. In a study using neural stem cell (NSC), MXene induced cytotoxicity at a relatively low concentration of 25 μg/mL [37]. Biocompatible MXenes are rising biomaterial that has recently received great attention for biological applications, with different biocompatibility reported depending on the cell type used in the evaluation, concentrations of MXene, and exposure time [41]. Therefore, in this study, we tried to investigate the biocompatibility of MXene using MSC, a stem cell, and the MTS results showed that MSC behavior is dependent on MXene concentration and has active cell viability under a concentration of 50 μg/mL.

**Figure 3:**
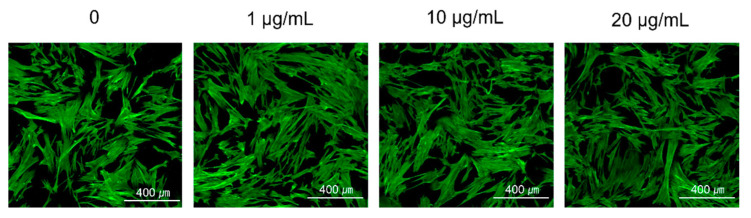
The CLSM image is the result of confirming the adhesion and proliferation of hMSCs.

Figure 3 shows the cell morphology on CLSM. All MXene conditions supported hMSC attachment, and the cells remained spread out. There was no significant difference in cell morphology with the MXene concentration.

**Figure 4:**
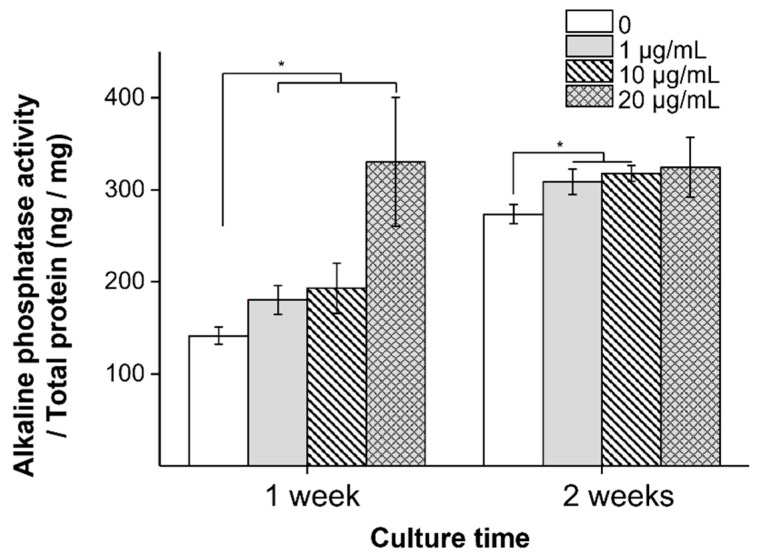
ALP activity of hMSCs for MXene concentration content after 7 days and 14 days incubation (* *p* < 0.05).

Alkaline phosphatase (ALP) activation was performed to confirm early osteogenic differentiation, where higher ALP activation indicates that osteogenic differentiation is being facilitated [42]. The ALP activity with MXene was assessed at 1 and 2 weeks without osteogenic differentiation (Figure 4). After 1 week, the ALP activity was higher with MXene than with the TCP control, and the maximum value was 20 μg/mL. After 2 weeks, despite the noticeable increase in ALP activity in the control, all MXene concentrations showed higher ALP activity than that of TCP. The ALP activity of cells cultured for 2 weeks was higher than after 1 week, indicating that MXene particles stimulate the initial osteogenic differentiation of hMSCs.

**Figure 5:**
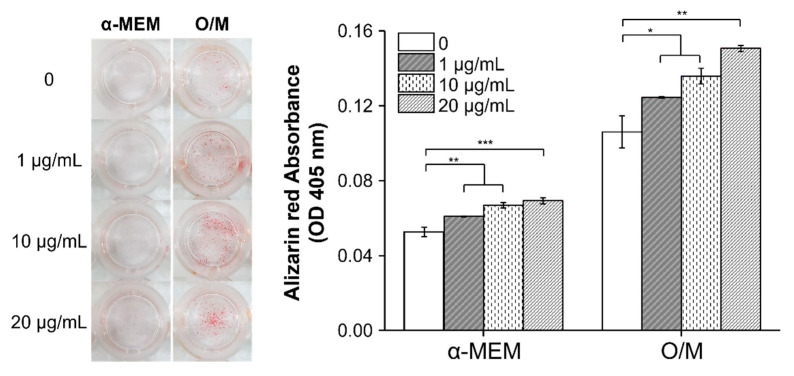
Alizarin red staining (ARS) showing the mineralization of cells cultured in normal and osteogenic media (* *p* < 0.05, ** *p* < 0.01 and *** *p* < 0.001).

Using ARS to analyze the bone mineral formation of cells and extracellular matrix, which indicates the last stage of osteogenic differentiation, after culture for 21 days, calcium deposits were visualized in cells and quantified with ARS staining (Figure 5). Cells treated with MXene showed greater osteogenic differentiation than with the TCP control [21]. The red part of the picture constitutes mineralized cells dyed by ARS. The calcium deposition caused by stem cell osteogenic differentiation was proportional to the MXene concentration and was higher in O/M medium than in α-MEM. The mineral deposition in hMSCs with MXene was significantly higher than with TCP.

**Figure 6:**
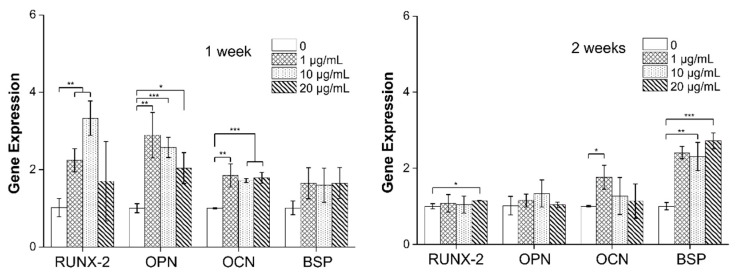
qRT-PCR after in vitro hMSCs culture to investigate the concentration of tissue culture plate and MXene treatment after 7 days and 14 days incubation. Early maker RUNX2 and OPN, middle maker OCN, late maker BSP (* *p* < 0.05, ** *p* < 0.01 and *** *p* < 0.001).

Figure 6 shows the results of qRT-PCR to examine osteogenic differentiation of hMSC with MXene at the mRNA level using the markers runt-related transcription factor 2 (RUNX-2) and osteopontin (OPN) (early), osteocalcin (OCN) (middle), and bone sialoprotein (BSP) (late), which are markers of the stages of bone differentiation in parentheses. After cell culture for 1 week, all MXene concentrations resulted in greater marker expression than TCP. Comparing the differences according to culture duration, the levels of the initial and middle stage markers (RUNX2, OPN, and OCN) were higher in the cells cultured for 1 week than at 2 weeks, whereas the levels of the late stage marker BSP was higher at 2 weeks. Comparing MXene concentrations, 20 μg/mL resulted in the strongest osteogenic gene expression. Interestingly, there was no difference in RUNX2 or OPN expression between MXene and TCP at 2 weeks. This demonstrates that the expression of early stage markers is induced very quickly by the MXene and decreases during more than one week of incubation; middle or late stage osteogenic markers are much more active in two weeks of incubation.

Several studies on toxicity and osteogenic differentiation induction for pre-osteoblast have been published before 2020, as MXene is expected to favor bone-tissue engineering due to its hydrophilicity and mechanical properties [16,20]. However, there are, yet, no reports of the effects of MXene particles on biocompatibility and osteogenic differentiation of mesenchymal stem cells (MSCs).

MSCs are multipotent stromal cells that can be differentiated into a variety of cells, such as osteoblasts, chondrocytes, myocytes (muscle cells), and adipocytes. Therefore, stem cells have the advantage of more clearly assessing the osteogenesis-inducing ability of biomaterials than studies using osteoblastic cell-line, which only have the potential of osteoblastic differentiation [43]. In addition, the practical healing of damaged tissue progresses slowly, so the use of stem cells, such as MSC, which can simulate an actual tissue environment, may obtain clearer data for developing biomaterials or biodevices that can enhance the ability to recover [44]. Hereby, we evaluated the effects of MXene on MSC osteogenesis in various ways. As a result, MXene can promote osteogenesis of MSC, but it is revealed that the differentiation level is dependent on MXene concentration and cell culture time.

These experimental data showed that Ti_3_C_2_ MXene has a significant effect on bone differentiation, which can provide useful information to many researchers on bone-tissue engineering. Starting with this study, studies should be followed to establish basic knowledge of MXene biomaterial by comparing and analyzing the biomedical characteristics between MXenes.

## 4. Conclusions

MXene particles with a stacked 2D-lamellar structure were intercalated, and their biomedical characteristics were evaluated by human mesenchymal stem cells. MXene concentrations >50 μg/mL were cytotoxic, while concentrations <20 μg/mL induced and accelerated osteogenic differentiation. These results provide information that MXene is osteoconductive and potentially applicable as a biomaterial for bone-tissue engineering.

## Data Availability

The data presented in this study are available on request from the corresponding author.
